# Cold-tolerant strains of *Puccinia striiformis* f.sp. *tritici* (*MLG1* and *MLG2*) persist under snow, triggering early epidemic of stripe rust in the Xinjiang region, China

**DOI:** 10.3389/fpls.2026.1742470

**Published:** 2026-02-11

**Authors:** Muhammad Awais, Haocheng Jiao, Xueqing Peng, Li Li, Sajid Ali, Jie Zhao, Zhensheng Kang, Jinbiao Ma

**Affiliations:** 1 State Key Laboratory of Ecological Safety and Sustainable Development in Arid Lands, Xinjiang Institute of Ecology and Geography, Chinese Academy of Sciences, Urumqi, China; 2 China-Tajikistan Belt and Road Joint Laboratory on Biodiversity Conservation and Sustainable Use, Xinjiang Institute of Ecology and Geography, Chinese Academy of Sciences, Urumqi, Xinjiang, China; 3 Xinjiang Key Laboratory of Conservation and Utilization of Plant Gene Resources, Urumqi, China; 4 State Key Laboratory for Crop Stress Resistance and High-Efficiency Production, College of Plant Protection, Northwest A&F University, Yangling, China

**Keywords:** early epidemics, overwintering survival, snow cover survival, temporal dynamics, wheat stripe rust

## Abstract

Global warming has changed snowfall patterns in recent years worldwide, which affected the timing and severity of wheat stripe rust outbreaks. In wheat-growing areas of Xinjiang, China, where cold temperatures prevail and snow typically accumulates after cultivation, stripe rust epidemics have traditionally emerged after snowmelt. However, the recently delayed snowfall in some regions has led to earlier-than-expected outbreaks. The role of spores of different *Puccinia striiformis* f.sp. *tritici* (*Pst*) genotypes surviving under snow remains largely unexplored. We performed *Pst* sampling at three stages in the Yili region of Xinjiang: (i) previous crop in summer May 2023, (ii) early infection before snowfall in the next winter cropping season during December 2023, and (iii) post-snowmelt sampling from the same crop in March 2024. We used 17 SSR markers to genotype 486 *Pst* isolates. Our findings revealed that snow survival enables cold-tolerant strains of *Pst*, contributing to the early stripe rust epidemics in the Yili region of Xinjiang in December 2023. Structure analysis revealed a similar genetic group across different seasons and before and after snowmelt populations. MLG analysis identified that MLG-1 and MLG-2 persisted from the summer 2023 crop, early winter seedlings before snow, and after snowmelt in the 2024 crop. While some multilocus genotypes, such as MLG_60, MLG_62, MLG_67, and MLG_83, are not viable under snow cover, the genetic diversity of *Pst* declined between summer and early winter crops (Simpson diversity = 0.8 to 0.7). The genetic diversity of *Pst* increased between before and after the snowmelt crop (Simpson diversity = 0.7 to 0.9). It suggested that a possible invasion of migratory genotypes or continued sporulation happened after infection beneath the snow. The cold-tolerant strains of *Pst* (MLG1 and MLG2) are responsible for the early epidemic in the Yili region of Xinjiang during seedling stage, where temperatures are below freezing and they can survive under snow covers in winter. *Pst* can also overlap with summer and winter crops to survive year-round in this region. Monitoring the migration pattern of these strains is crucial as it causes disease in wheat seedlings, leading to more crop losses compared to other lineages that infect mature crops.

## 1 Introduction

Plant disease epidemic regions are undergoing transformations because of shifting global climate patterns, including altering temperature, precipitation rate, and snowfall time (Skripnuk and Samylovskaya, 2018; Hossain et al., 2024). These changes stimulate disease epidemics and facilitate the emergence of new pathogen races and the migration and adaptation of exotic pathogen races in new regions. These new races may break down resistant genes in host plants (Newbery et al., 2016; Velasquez et al., 2018; Garrett et al., 2006). In the context of *Puccinia striiformis* f.sp. *tritici* (*Pst*), the causal agent of wheat stripe rust, environmental changes associated with climate variability directly impact overwintering survival, spore dispersal, and infection cycles (Awais et al., 2025; Zhang et al., 2025). Warmer winters and delayed snowfall may shorten the pathogen dormancy periods (Singh et al., 2023). It exemplified in higher snow blight (*Phacidium infestans*) infections in pine trees due to early snowmelt (Barbeito et al., 2013). Previous studies demonstrated that *Puccinia striiformis* f.sp. *tritici* (*Pst*) mycelia within living leaves can be killed by low temperatures independently of host senescence (Ma et al., 2015). However, the role of cold stress in shaping *Pst* populations and genetic diversity under natural conditions remains poorly understood. Temperature extremes in both summer and winter limit pathogen survival, as *Pst* cannot persist in extremely hot or extremely cold environments (Lyon and Broders, 2017). Generally, the optimal temperature for spore germination ranges from 8°C to 12°C, with an upper limit of approximately 20°C (de Vallavieille-Pope et al., 1995). In cold wheat-growing regions, snow exerts a dual influence on stripe rust epidemiology. While it provides protection and sustains the pathogen during harsh winters, facilitating early-season epidemics, it also acts as a selective filter, shaping the genetic structure of *Pst* overwintering populations. The severity of wheat stripe rust has significantly escalated in cold regions over the past few decades primarily due to global warming (Zhang et al., 2024), which was noticed in the Central Asian region, Uzbekistan, where the clonal lineage of *Puccina striiformis* causes a countrywide epidemic (Awais et al., 2025).

In China, the Xinjiang epidemic region is important because of several bordering countries with hot- and cold-adopted *Pst* epidemic regions, including Kazakhstan, Kyrgyzstan, Tajikistan, Afghanistan, Pakistan, India, Russia, and Mongolia. This geographic position makes the region highly vulnerable to incursions of foreign *Pst* races (Awais et al., 2025), thereby increasing the risk of wheat stripe rust epidemics. The region is also surrounded by major mountain ranges, the Tianshan mountains in the center, the Altai mountains to the north, and the Kunlun mountains to the south, which contribute to population subdivision by acting as natural barriers (Awais et al., 2023). In addition to these geographical factors, Xinjiang is characterized by a vast diversity of climatic conditions. Summers in the lowland basins can exceed 40°C, while winters in mountain valleys commonly reach –20°C to –30°C, creating strong ecological gradients that influence both host and pathogen dynamics. In recent years, Xinjiang has also been strongly affected by climatic change. Rising temperatures compared with those of previous decades have altered snowfall patterns and intervals, directly influencing both disease epidemiology and cropping schedules (Chaloner et al., 2021). These shifts not only affects wheat growth cycles but also modifies the conditions under which *Pst s*urvives and initiates infection, thereby increasing the risk of unexpected stripe rust epidemics.

Despite the climatic change, the emergence and evolution of *Pst* races pose a similar challenge. The high evolutionary potential of *Pst*, driven by mutations, recombination, and migration, enables the pathogen to rapidly overcome host resistance and adapt to changing environments (Schwessinger, 2017). Genetically uniform crop monocultures and high planting density in modern agriculture have accelerated the emergence of virulent *Pst* races capable of overcoming disease-resistant crop varieties and promote the pathogen’s population size and genetic diversity (Singh et al., 2023). This constant turnover of virulent races undermines the durability of resistant cultivars and complicates breeding programs aimed at long-term disease control. Consequently, monitoring race evolution and understanding the mechanisms underlying *Pst* adaptability remain critical for the sustainable management of stripe rust under present and future climatic conditions. In this context, investigating before snow and after snow populations in Xinjiang provides an opportunity to clarify how climatic variability shapes *Pst* diversity, lineage survival, and the timing of epidemics.

Snow covering wheat regions in Xinjiang is highly significant for the survival, evolution, and spread of *Pst* in China. Snow survival creates conditions for the long-term persistence of *Pst* lineages while, at the same time, immigration of exotic spores after snowmelt may introduce new diversity in this region. Given China’s vast climatic heterogeneity, from cold northern wheat-growing regions to mild southern environments, cold tolerance may represent a critical, yet overlooked, factor shaping the long-term persistence and spread of *Pst*. Investigating whether certain lineages exhibit enhanced cold tolerance will help clarify their evolutionary dynamics, regional adaptability, and implications for stripe rust epidemiology under both current and future climate scenarios.

The ability to predict pathogen survival potential may enable the timely implementation of integrated disease management strategies to reduce inoculum sources and prevent the formation of disease foci.

## 2 Materials and methods

### 2.1 Wheat rust surveillance

Wheat rust surveillance was conducted across multiple regions of Xinjiang in May to June 2023 in different locations of Yili following the protocol of Ali and Hodson (2017). Surveillance was again repeated in different parts of Xinjiang (Zhaosu, Tekes, Gongliu, Yining, and Xinyuan) in December on the next winter crop at different parts in 2023, and early infection samples before snow were obtained in Xinyuan and Gongliu of Yili region, while the other regions of Xinjiang wheat fields were covered already with snow before the seedlings come out. Only Xinyuan and Gongliu were spotted with early infection, and these regions’ infected wheat was covered with snow in mid-December. At the same time, the fields in other regions were already covered with snow before seedling germination, which delayed early disease establishment. Following snowmelt, surveillance was repeated in the same regions in 2024 to collect disease samples that had survived under snow. These post-snow samples were then compared with samples from the previous year to assess the overwintering survival and persistence of the pathogen.

### 2.2 Sample recovery and genotyping

Infected leaf samples with single lesions were collected from fields, which were used to extract DNA following the CTAB method (Ali et al., 2017). The quality of DNA was determined using a spectrometer (NanoDrop 1000, Thermo Scientific, Waltham, MA, USA). The DNA was stored at -20°C for later use. A total of 17 SSR primers were selected for genotyping *Pst* isolates from before and after snow crop (**Supplementary Table S1**). PCR amplication was carried out using high-quality reagents, including Tag Plus DNA Polymerase (Sangon, B600090), 10× PCR buffer (with Mg^2+^; Sangon, B600017), dNTPs (10 mM; Sangon, B500056), sterilized deionized water (E607017), 6× DNA Loading Dye (ThermoFisher, R0611), DNA Ladder Mix (100–3,000 bp; B500437), 50× TAE (Sangon, B548101), Agarose H (Sangon, A500016), 1× TE (Sangon, B548106), POP-7TM Polymer (ThermoFisher, 4363785), and HiDi™ Formamide (ThermoFisher, 4311320) (**Supplementary Information**: **Supplementary Tables S2**, **S3**). A standardized PCR protocol was followed, and the PCR products were then detected through a 3730xl ABI sequencer.

### 2.3 Population genetics analysis

Raw SSR data were compiled into an Excel sheet and subsequently underwent various population genetic analyses. The population genetic structure was estimated using the STRUCTURE 2.3.4 program (Pritchard et al., 2000), employing the model-based Bayesian clustering method. A Markov chain Monte Carlo (MCMC) simulation was conducted within the Bayesian framework. The assignment of individuals to different clusters was performed from K1 to K10, with each value undergoing 10 independent runs with 100,000 iterations and a burn-in period of 100,000. CLUMPAK (cluster Markov packager across K) was used to investigate multiple separate runs for each fixed value of K and distinguish among runs belonging to substantially different clusters and got the best k value (Kopelman et al., 2015). Various population genetic diversity parameters, including Simpson diversity, Shannon diversity, evenness, and multilocus genotypes (MLG), were estimated using the Genodive program (Meirmans, 2020). Principal coordinate analysis (PCoA) was conducted in R using the Adegenet package (Jombart et al., 2010). A phylogenetic tree was constructed using Population software (Langella, 2002), employing Nei’s genetic distance. The *F_ST_* value was calculated using the POPPR R package. A migration network based on effective migrants (Nm, D) was visually generated to illustrate the gene flow pattern among seasonal (summer and winter) and pre- and post-snow samples using the diversity package in R (Bai et al., 2021).

## 3 Results

### 3.1 Microsatellite marker profiling for genotyping

A total of 17 SSR markers were used to genotype the 486 isolates from summer crop season 2023, winter crop season 2023 before snowfall, and same crop season after snowmelt period 2024. These markers were previously used as well in overall China *Pst* population genetic study (Awais et al., 2022). In this study data set, one marker—NRJN12—exhibited monomorphism, while the remaining 16 showed polymorphism. Markers NRJO27, NRJN11, NRJ13, and NRJN2 displayed the highest number of alleles, ≥6. These marker profiling results (**Table 1**) indicated that these markers are suitable for conducting a population genetic analysis (**Supplementary Information**: **Supplementary Figure S1**).

**Table 1:** Microsatellite markers profiling used to conduct the genotyping of *Puccina striiformis* isolates from across crop seasons, before snow, and after snowmelt in the Xinjiang region of China.

Locus	Number of alleles	Average alleles	Ho	Hs
NRJN12	1	1	0	0
NRJN8	5	1.01	0.009	0.01
NRJN13	6	1.026	0.02	0.025
NRJN3	3	2.01	0.961	0.503
NRJN11	7	2.097	0.988	0.523
NRJO27	12	1.182	0.057	0.155
NRJN6	4	1.021	0.007	0.021
NRJO21	5	1.061	0.044	0.058
NRJN10	4	1.073	0.053	0.069
NRJO18	5	2.029	0.959	0.507
NWU6	3	1.017	0.017	0.016
NRJO20	5	1.059	0.041	0.056
NRJN2	8	1.396	0.227	0.285
NRJN4	5	2.572	0.945	0.612
NRJN9	2	1.008	0.008	0.008
NRJN5	4	1.038	0.036	0.037
NWU12	3	2.037	1	0.509
Overall	4.824	1.39	0.316	0.2

### 3.2 Temporal shift in the genetic diversity of *Pst* linked to snow cover and seasons

We have collected 127 samples from the last crop summer season 2023 (LC), 279 samples from the next winter season in December before snowfall (LCBS), and 80 samples from the same season crop after snowmelt (AS). Our results showed high gene diversity (HS) in the LC crop season (HS = 0.21), with a high average number of alleles (3.47) compared with other temporal populations of Xinjiang. However, overall, this range is considered low when compared with the results of previous studies of *Pst* populations in China (Awais et al., 2022). The gene diversity and average number of alleles decreased in the next crop season before snowfall (0.178 and 2.76, respectively) (**Table 2**). However, among the before snow and after snow samples, gene diversity was found to be increased (0.18 to 0.21), while the number of alleles was comparatively slightly low after the snow sample (2.58).

**Table 2:** Genetic diversity of *Puccinia striiformis* across crop seasons, before snowfall and after snowfall, in the Xinjiang region of China.

Population	Num	Eff_num	Ho	Hs	Gis
LC	3.471	1.397	0.313	0.212	-0.474
LCBS	2.765	1.369	0.298	0.178	-0.676
AS	2.588	1.417	0.337	0.209	-0.613

LC, last summer crop 2023; LCBS, last crop before snow (winter crop 2023); AS, crop after snowmelt 2024; Num, number of alleles observed; Eff_num, effective number of alleles; Ho, observed heterozygosity; Hs, expected heterozygosity within populations.

In comparison to the last crop summer season (2023) and the next crop season before snowfall, the highest genetic diversity was observed in the last crop season (2023). Simpson diversity was 0.83, and Shannon diversity was 1.525. Conversely, the genetic diversity in the next crop before snow was lower, with a Simpson diversity of 0.7 and a Shannon diversity of 0.906. The multilocus genotypes (MLG) also decreased from LC (53) to LCBS (36).

In comparison to before snowfall and after snowfall samples, the genetic diversity exhibited an increasing trend. The highest genetic diversity was observed in the after snowmelt crop with Simpson diversity (Diu) equal to 0.89 and Shannon diversity (Shc) equal to 1.40 (**Table 3**). However, the MLGs slightly decreased in the after snowmelt samples.

**Table 3:** Clonal diversity of *Puccinia striiformis* populations across crop seasons, before snowfall, and after snowfall in the Xinjiang region of China (2023–2024).

Population	Size	MLG	eff	div	diu	eve	shc	shu
LC	127	53	6.196	0.845	0.839	0.117	1.525	1.209
LCBS	279	36	3.342	0.703	0.701	0.093	0.906	0.785
AS	80	31	9.756	0.909	0.898	0.315	1.402	1.207
Average	162	40	6.432	0.819	0.812	0.175	1.278	1.067

LC, last summer crop 2023; LCBS, last crop before snow (winter crop 2023); AS, crop after snowmelt 2024; MLG, number of genotypes found in populations; eff, number of effective genotypes; div, Simpson’s diversity index clone corrected; diu, Simpson’s diversity index clone uncorrected; eve, evenness value; Shc, Shannon index (shc) clone corrected; shu, Shannon–Weaver index uncorrected clone.

### 3.3 Genetic structure of *Pst* across seasons and snow periods

**Figure 1:**
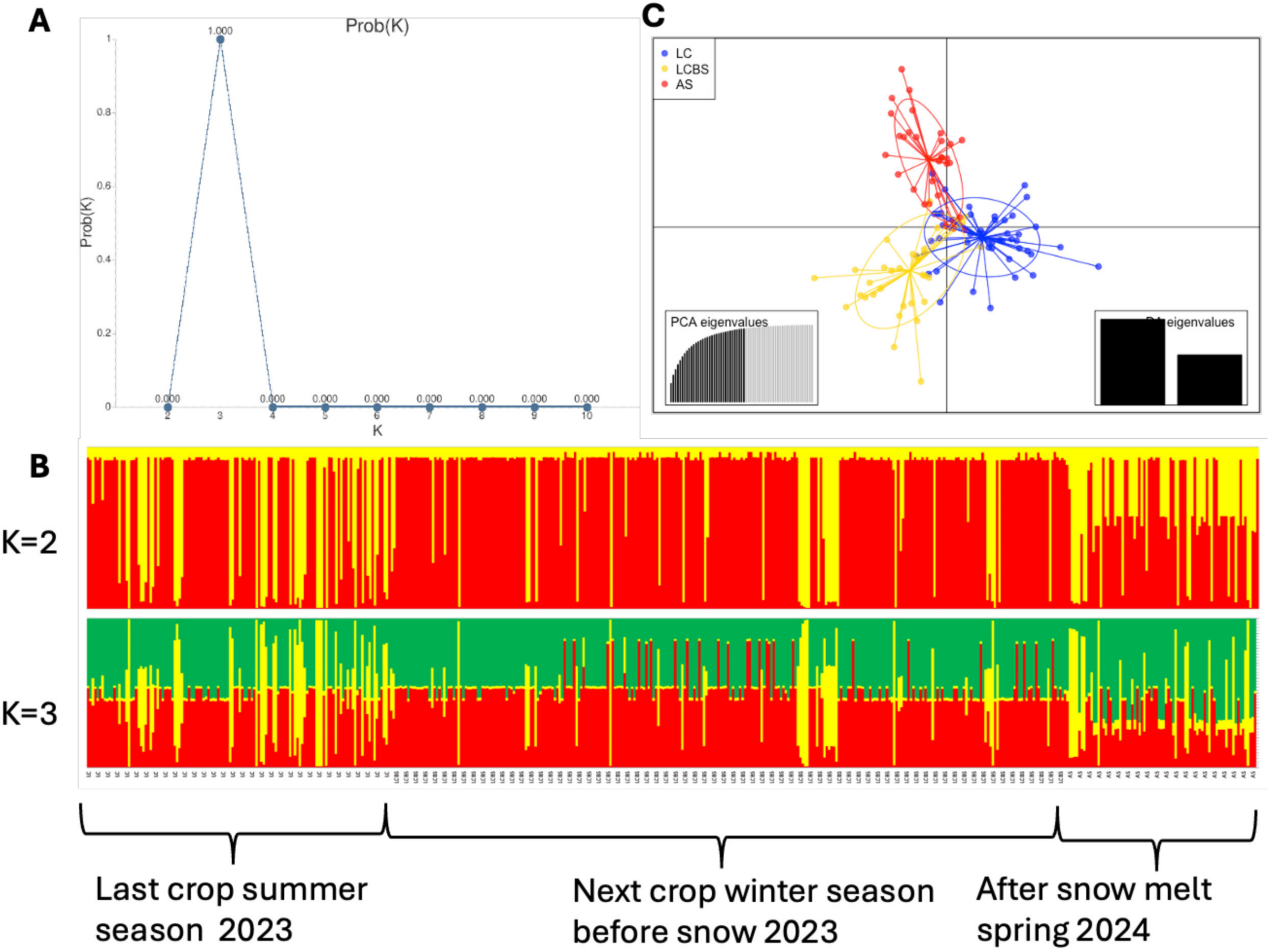
**(A)** Temporal pattern of *Puccinia striiformis* population genetic structures across different crop seasons and understanding the effect of snowfall on the survival of different genotypes. Best K value based on Structure harvester. **(B)** Cluster groups based on Structure output (K-2 to K3). Red color, G1; yellow, G2; green color, G3. **(C)** PCA plot analysis on POPPR R software.

The temporal dynamics (spring wheat crop in 2023 season in May 2023, next early winter wheat crop in December 2023 before snowfall, and same winter wheat crop after snowmelt in March 2024) of the *Puccinia striiformis* population structure were analyzed using the Structure software. We determined the optimal K from K-2 to K10 using CLUMPPAK, which give the optimal value at K-3. At K-2 (**Figure 1A**), two distinct genetic groups were identified. Group G1 was predominant in the 2023 spring crop season (**Figure 1B**). Interestingly, this group overlapped with group G2 in the early seedling stage of the following winter crop season, before the snowfall in December 2023. Later, group G1 persisted in the same crop after snowmelt in March 2024. In contrast, group G2 exhibited limited genetic differentiation across all three temporal dynamics. At K-3, no distinct population structure groups were found. All three groups showed admixture in all three temporal dynamics. The results in this region indicate that the same population group overlaps during both winter and summer. Notably, this group survives under snow during December to March in the Yili region of Xinjiang.

The result was further validated through PCA plot analysis (**Figure 1C**), where three of the temporal group populations overlap each other, suggesting gene flow among three temporal groups.

### 3.4 Local survival vs. migrants of Pst across snow cover and seasonal transitions

**Figure 2:**
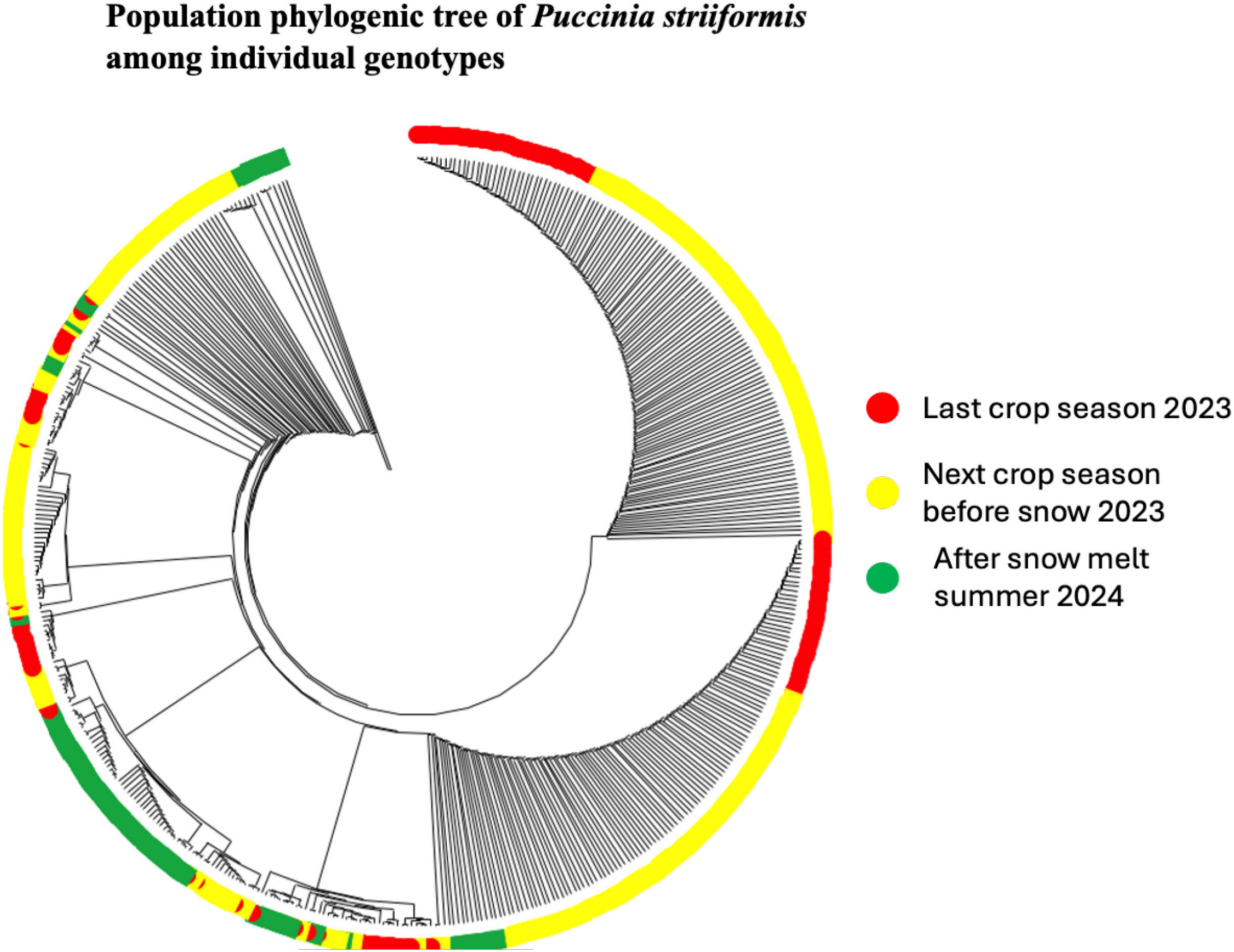
Phylogenetic tree of *Puccinia striiformis* population during different temporal factors (last crop summer season 2023, next crop winter season before snow 2023, and after snowmelt summer 2024). The phylogenic tree was constructed using Nei’s genetic distance.

**Figure 3:**
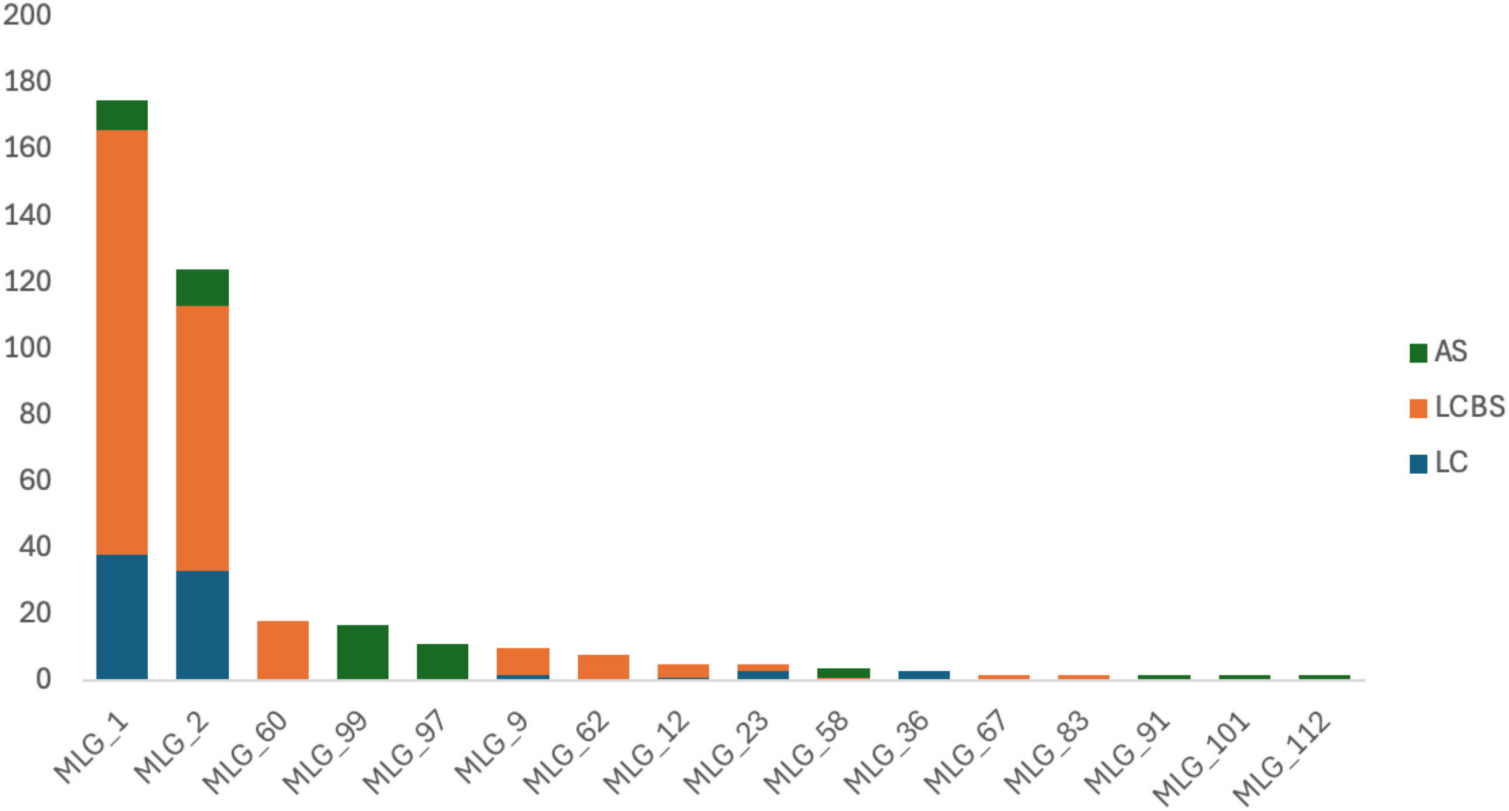
MLG distribution of *Puccinia striiformis* population during different temporal dynamics (last crop summer season 2023, next crop winter before snow 2023, and after snowmelt in March 2024). Single MLGs that found only one time were filtered out from data sets, only MLG that found more than two times were considered for MLG analysis.

To evaluate the potential for the local survival of *Puccinia striiformis* genotypes in Xinjiang, we performed STRUCTURE (**Figure 1B**), DAPC (**Figure 1C**), phylogenic tree analysis (**Figure 2**), and multilocus genotype assessments (**Figure 3**). These approaches provided strong evidence that certain genotypes are capable of persisting throughout the year, highlighting their role in sustaining the pathogen population across growing seasons and pre-snow fall and after snowmelt time, which was further validated through MLG analysis. MLG1 and MLG2 were detected in the sample taken during the last crop summer season (2023). These two MLGs were found as well in the last crop before snow (LCBS) and after snowmelt (AS) population, suggesting high tolerance to cold temperature and being well adapted to this cold epidemic region. Additionally, MLG_36 was exclusively found in the last crop summer season, which was not overwintering. MLG_60, MLG_62, MLG_12, MLG_67, and MLG83 were only present in the samples taken before snowfall (LCBS). They were not detected in the last crop summer season (2023). This suggests that these genotypes may have migrated from other regions or emerged through sexual reproduction. However, these genotypes cannot withstand snowfall and were eliminated after snowfall.

On the other hand, some genotypes, such as MLG_23 and MLG_9, were found in the last crop and were also found in the next crop season, but these were not found after snowmelt. However, MLG_99 and MLG_9 were found in a very limited number after snowfall, while they were absent before snowfall. This indicates that these genotypes may come through migration (**Figure 3**).

### 3.5 Estimating gene flow across cropping seasons and snowing periods

**Figure 4:**
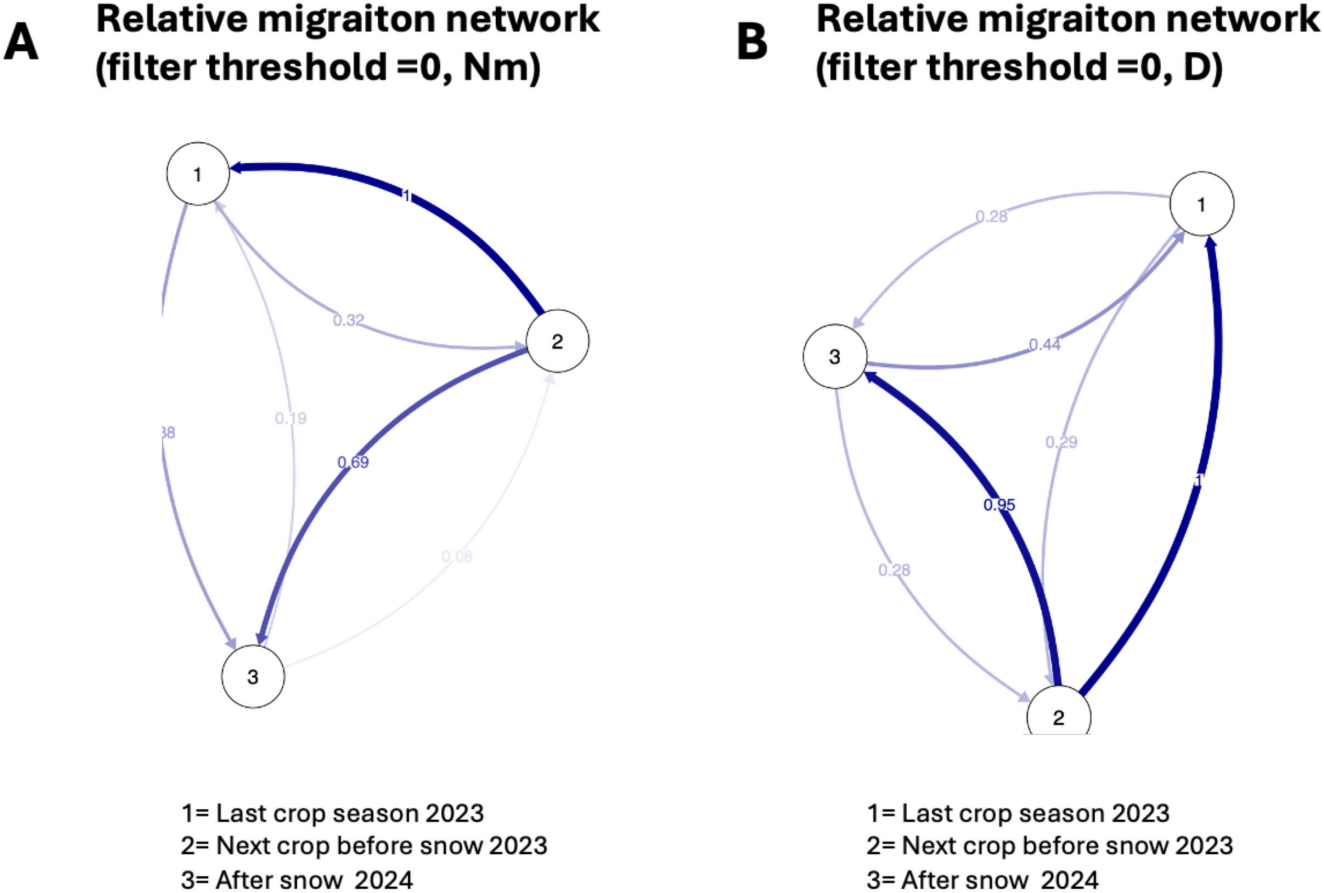
Relative migration network of *Puccinia striiformis* population during different temporal dynamics (last crop summer season 2023, next crop winter before snow 2023, and after snowmelt in March 2024). **(A)** Relative migration network of Nm value. **(B)** Relative migration network of Jost’s **(D)** The line thickness describes the strength of gene flow among populations.

**Figure 5:**
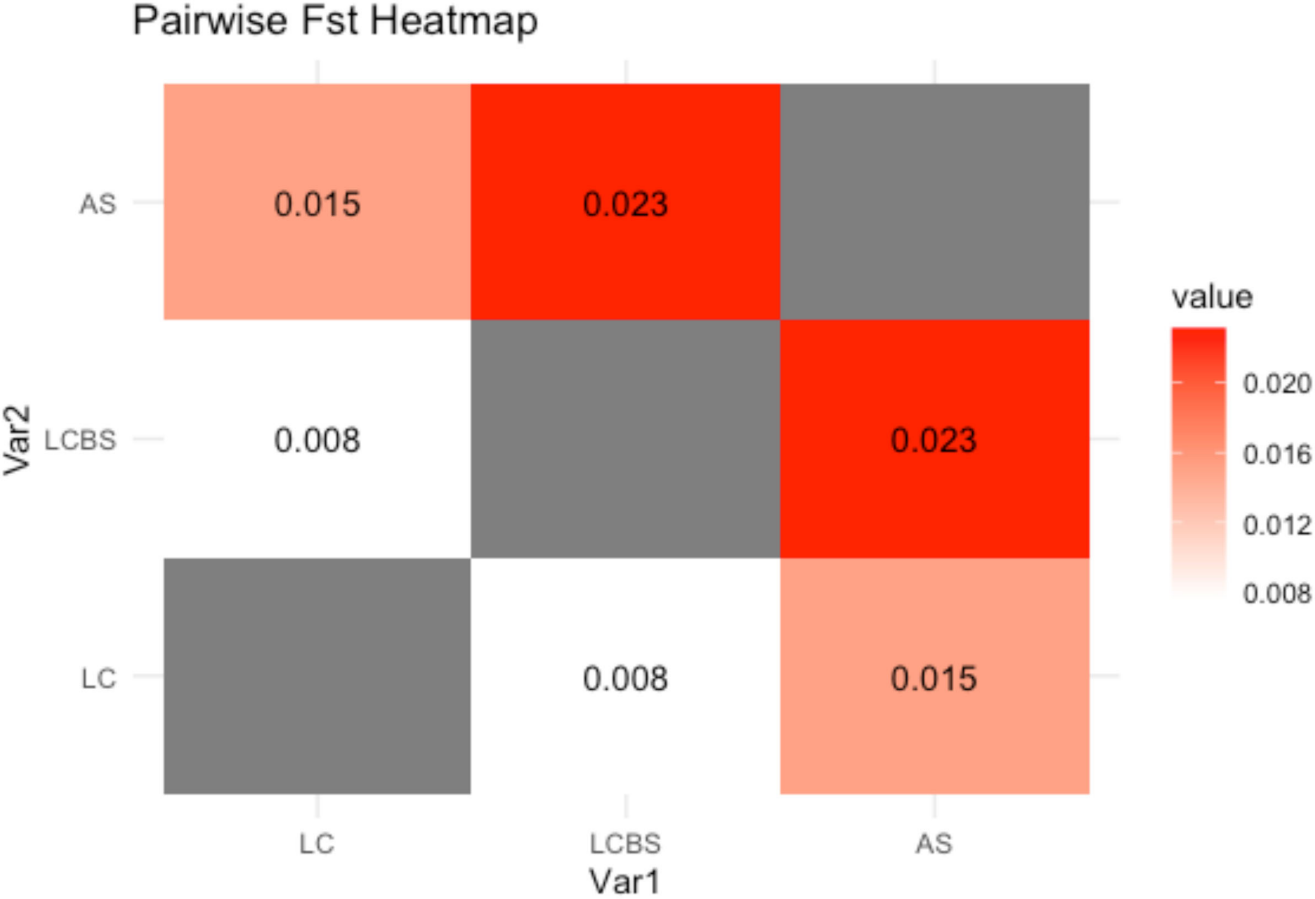
*F_ST_* value of *Puccinia striiformis* population in different cropping season and snowfall periods (summer 2023, early winter 2023 before snowfall, and same crop after snowmelt 2024).

Gene flow during cropping seasons and pre- and post-snowmelt periods was estimated using a relative migration network (Nm, D; **Figure 4**). Our results showed that the *Pst* population in the last crop summer season (2023) and the next crop early winter before snowfall (2023) had high gene flow, as reflected in the *F_ST_* value (**Figure 5**). The *F_ST_* value between the last crop summer season and the next crop early winter before snowfall (2023) had the lowest *F_ST_* value (0.008). On the other hand, comparing the samples before and after snowfall periods showed high gene flow, suggesting the high survival of genotypes under snow covers. This result was supported by the lowest F_ST_ value (0.023). However, when comparing the last crop summer season (2023) and the after snowmelt period (2024), we noticed a weak gene flow.

## 4 Discussions

*Puccinia striiformis*, a fungal pathogen, causes stripe rust epidemic in various regions and poses a significant threat to global food security (Chen, 2005; Hovmøller et al., 2011). Generally, *P. striiformis* exhibits sensitivity to temperature. A study conducted by de Vallavieille-Pope (2018) using various isolates of the warrior race from Europe reported that none of the infected individuals exhibited any noticeable symptoms when the temperature exceeded 23°C. However, due to its high evolutionary potential (Riella et al, 2024), some genotypes of *P. striiformis* have emerged with adaptability against high temperatures (Zhang et al., 2024). Among different ecological factors, snowfall plays an important role in stripe rust disease epidemiology. However, due to climatic change, the snowfall pattern is also changed, which may affect the disease epidemic occurrence time and disease severity. As observed in the epidemic region of Xinjiang, China, this was due to late snowfall. The disease occurs early in December 2023 in the Yili region of Xinjiang. Based on previous population genetic studies, different epidemic zones have been reported worldwide. Some showed a distinct population genetic structure compared to others (Ali et al., 2014; Thach et al., 2016). Moreover, a migration pattern was observed among those epidemic regions, which have identical topography, similar environmental conditions, and an availability of susceptible host and exchange of winds flows. However, due to climate change, the population genetic structure may be reshaped in various epidemic regions, potentially favoring the migration of exotic lineages to establish their populations in new regions and causing early epidemics in some parts.

**Figure 6:**
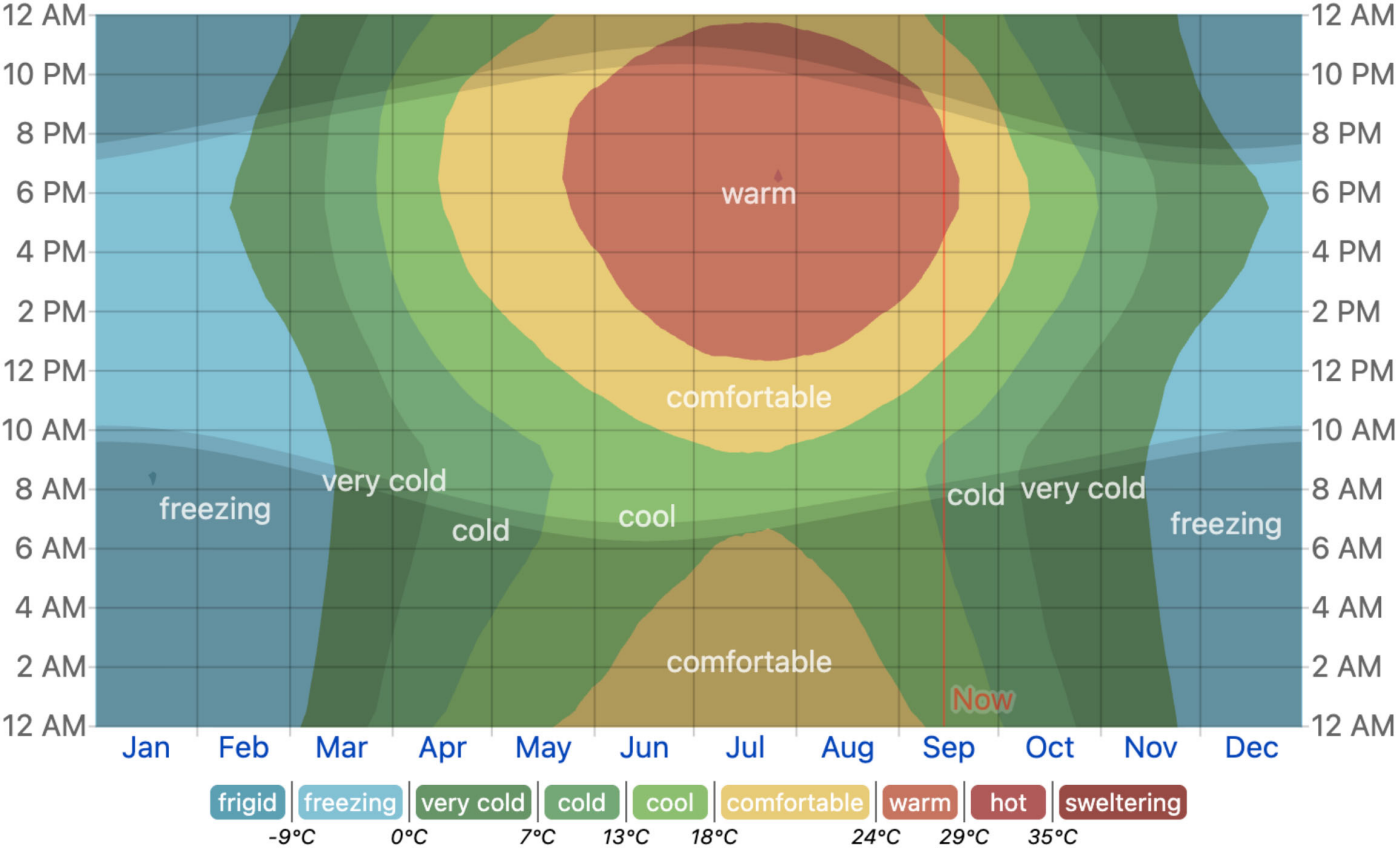
Average temperature in Yilli, Xinjiang region (the image was generated through online weather data from the website www.weatherspark.com). .

China hosts the world’s largest epidemic region of wheat stripe rust, characterized by a significant divergence in the *Pst* population structure. These epidemic zones exhibit distinct environmental characteristics, varying cropping patterns, and differing host availability (Ma et al., 2016). Among the different epidemic regions in China, Xinjiang stands out due to its geography, diverse ecological zones, and *Pst* epidemic characteristics. Based on the annual weather forecast of this region (**Figure 6**), it can be referred to as the cold *Pst* epidemic region. Typically, disease epidemics occur in this area during spring after snowmelt. Seedlings are primarily protected under snow, which occurs immediately after cultivation. However, recent climatic changes have disrupted this epidemic cycle. Disease has occurred earlier in the Xinjiang region of Yili in December 2023, while other regions have experienced delayed epidemics during spring when snow melts. However, the snow pattern has a bit changed, but the overall temperature in this region in winter is still freezing (below 0°C; **Figure 6**). Our results identified the cold-tolerant strains MLG_1 and MLG_2, which did not only exhibit cold tolerance but also survived under snow cover. Additionally, these genotypes demonstrated the ability to jump on summer and winter crops to survive the year-round cycle in Yili. As we have observed, this region’s summer is not even as hot compared to other parts of China, which may have helped these genotypes to survive even in summer. Li and Zeng (2002) reported that *Pst* could successfully survive in winter, even when the monthly mean temperature is below −10°C, as long as the wheat seedlings are covered with snow. We observed a low F_ST_ value among summer 2023 crops and the next early winter crop in December before snowfall occurred. Moreover, these two-crop season has high gene flow. In China, *Pst* can complete a year-round cycle in the northwest and southwest, significantly impacting the main wheat production region in the east (Awais et al., 2023; Chen et al., 2014). In the Yili region of Xinjiang, both spring and winter wheat crops are cultivated. During wheat stripe rust surveillance in this region, we noticed that this region has high disease severity compared to those in other parts of Xinjiang. As previous studies have reported, the regions where both spring and wheat crops are cultivated experience a more severe epidemic than those regions with either spring or winter wheat crop grown in the same season (Chen, 2005). Furthermore, in terms of adaptability to low temperatures, wheat stripe rust can emerge very early in the seedling stage, resulting in more severe damage in that area (Chen, 2005).

**Figure 7:**
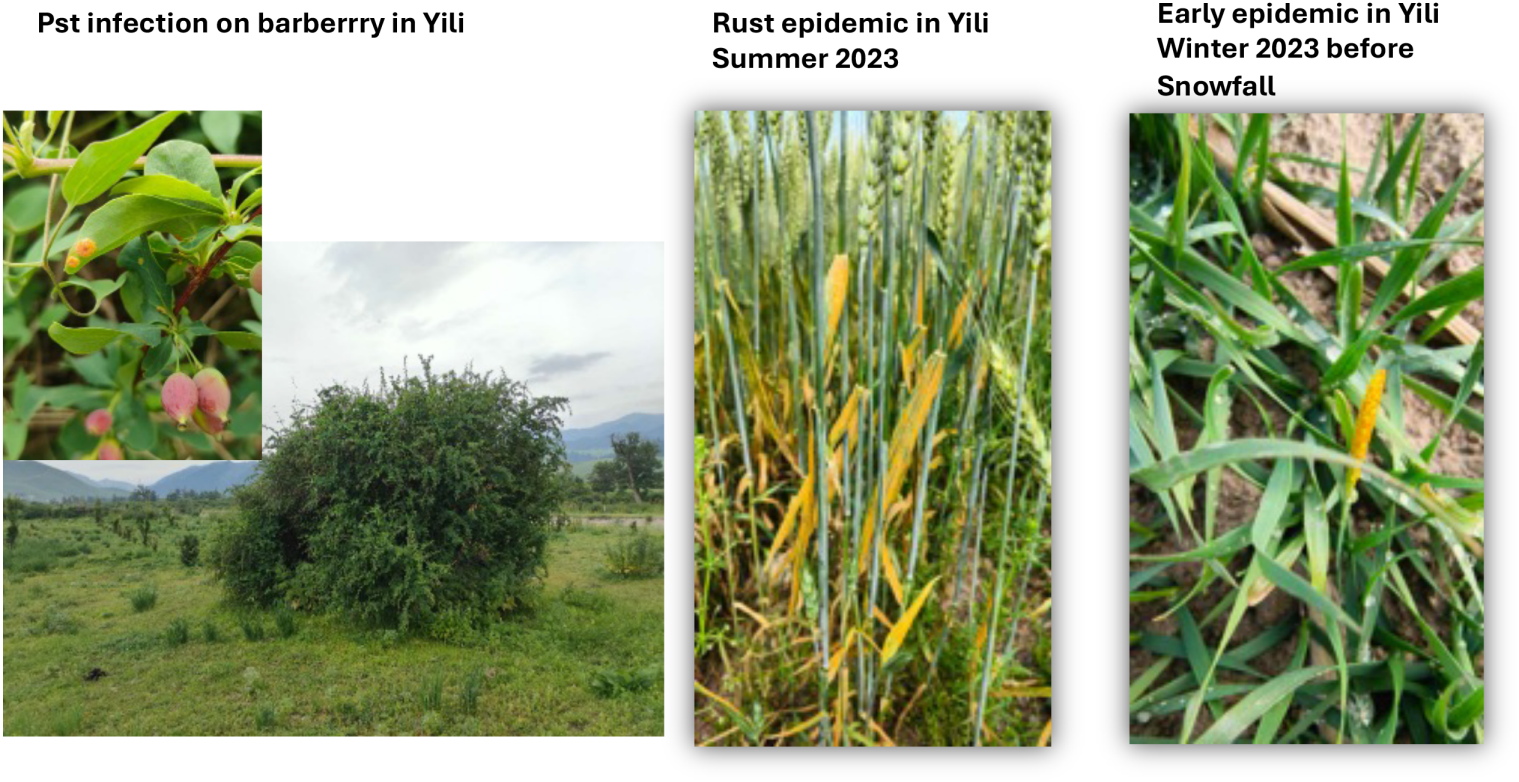
Early stripe rust epidemic in Yili, Xinjiang region, with infections on *Barberis* species facilitating the emergence of new races in the area.

However, some genotypes did not survive during snow covers and low temperature, i.e., MLG_60, MLG_62, MLG_67, and MLG_83. As reported by Zhang et al. (2025), in winter, no stripe rust disease was reported in the east of Inner Magnolia and north of Heilongjiang, where the regions’ winter is too cold for *Pst* to cause infections. Interestingly, these genotypes did not survive during snow cover. They were also not found in the last summer crop 2023, suggesting that these genotypes may be migrant and may have come from neighboring regions as our recent work showed the potential of migration of the *Pst* population in this region from Central Asian countries (Awais et al., 2025) or of emerging through sexual reproduction on alternative host barberry, which was commonly found in this region to be with disease infection (**Figure 7**).

Monitoring cold-tolerant *Pst* strains is crucial for effective disease management. These strains survive harsh winters, initiating infections earlier, often at the seedling stage. Early epidemics accelerate the disease cycle and increase the risk of widespread infections throughout the cropping season. Seedling-stage outbreaks cause more severe wheat damage and yield loss, posing a greater threat to regional food security than late-season *Pst* epidemics. Understanding cold-tolerant populations and their spread is essential to develop timely monitoring systems, breeding-resistant cultivars, and sustainable management practices.

## 5 Conclusions

We investigated the impact of delayed snowfall due to climate change on the stripe rust epidemic and their population genetic diversity in Xinjiang epidemic region of China. We also explored genotype overlaps during summer and winter crop seasons. Recently, an unusual early stripe rust epidemic occurred in the Yili region of Xinjiang, where seedlings were generally protected by snow until spring in the previous years. However, delayed snowfall in the 2023 snow season led to the first earlier epidemic in the region. The region’s winter temperature dropped to -25°C, which was generally unfavorable to cause disease. However, in this study, we identified cold-tolerant strains of *Pst*, MLG_1 and MLG_2, that have the potential to cause infection in low temperatures and can survive under snow cover. We noticed pathogen population overlaps on summer and winter crop seasons, completing the disease cycle. However, genetic diversity decreased in the region. Some multilocus genotypes were observed in winter crops but not in summer crops, suggesting migration from other regions since this region has connectivity with central Asian countries and Pakistan, where both cold and hot *Pst* epidemics are present. Monitoring the migration of these cold-tolerant strains of *Puccinia striiformis* is crucial as they can infect wheat seedlings in winter, causing more crop damage than infection during the crop’s mature stage.

## Data Availability

The original contributions presented in the study are included in the article/**Supplementary Material**, further inquiries can be directed to the corresponding author/s.
