# Supplementary material for: Cold-tolerant strains of Puccinia striiformis f.sp. tritici (MLG1 and MLG2) persist under snow, triggering early epidemic of stripe rust in the Xinjiang region, China
Source: Front Plant Sci. 2026 Feb 11;17:1742470. doi: 10.3389/fpls.2026.1742470 (PMC12932588; doi:10.3389/fpls.2026.1742470)
Supplement: Supplementary file 1 [file DataSheet1.docx]

**Supplementary information:**

**Table S1 Seventeen sets of simple sequence repeat (SSR) markers used for genotyping of *Puccinia striiformis* f. sp. *tritici* populations of Xinjiang, China.**

| **Marker** | **F (5'-3')** | **Florescent adaptor** |
| --- | --- | --- |
| RJN12-F | TGTTGACAAACACGACGACC | 5`HEX |
| RJN8-F | ACTGGGCAGACTGGTCAAC | 5`6-FAM |
| RJN13-F | TTAGCTCAGCCGGTTCCTC | 5`HEX |
| RJN3-F | TGGTGGTGCTCCTCTAGTC | 5`NED |
| RJN11-F | CCGACACCTCCTCTGATCG | 5`HEX |
| RJO27-F | CGTCCCGACTAATCTGGTCC | 5`6-FAM |
| RJN6-F | CAATCTGGCGGACAGCAAC | 5`NED |
| RJO21-F | TTCCTGGATTGAATTCGTCG | 5`HEX |
| RJN10-F | ACGTGCCAGCTCAACTCTC | 5`6-FAM |
| RJO18-F | CTGCCCATGCTCTTCGTC | 5`NED |
| WU6-F | CAGCTCTGTTGATTTCTTCC | 5`HEX |
| RJO20-F | AGAAGATCGACGCACCCG | 5`6-FAM |
| RJN2-F | TTGTGGCGGAAGGGAACG | 5`HEX |
| RJN4-F | CATTCATGACCCTCGCCTC | 5`6-FAM |
| RJN9-F | TTAGGCGCTCAACAAGCAG | 5`NED |
| RJN5-F | AACGGTCAACAGCACTCAC | 5`6-FAM |
| WU12-F | GGAAACTGTAGCACCTTCAC | 5`NED |

**Table S2 PCR preparation for *Puccinia striiformis* isolates collected from across crop season, before snowfall and after snowfall in this Xinjiang region of China.**

| **Component** | **Concentration** | **Volume (**μL**)** |  |
| --- | --- | --- | --- |
| Template DNA | 20-50 ng/μL | 1.0 |  |
| Primer F | 10 μM | 0.5 |  |
| Primer R | 10 μM | 0.5 |  |
| dNTPs（mix） | 5 μM | 0.5 |  |
| Taq Buffer（with MgCl_2_） | 10 X | 2.5 |  |
| *Taq* enzyme | 5 U/μL | 0.2 |  |
| Add ddH_2_O to |  | 25 |  |

**Table S3 PCR amplification program for *Puccinia striiformis* isolates collected from across crop season, before snowfall and after snowfall in this Xinjiang region of China.**

| **No** | **Reaction** | **Temperature and cycle** | **Time duration** |
| --- | --- | --- | --- |
| 1 | Predenature | 95°C | 5 min |
| 2 | Denature | 94°C | 30 s |
| 3 | Annealing | 60°C with decreasing 0.5°C per cycle | 30 s |
| 4 | Extension | 72°C | 30 s |
| 5 | 2-4 cycles | 10 cycles |  |
| 6 | Denature | 94°C | 30 s |
| 7 | Annealing | 55°C | 30 s |
| 8 | Denature | 72°C | 30 s |
| 9 | 6-8 cycles | 30 cycles |  |
| 10 | Final extension | 72°C | 10 min |


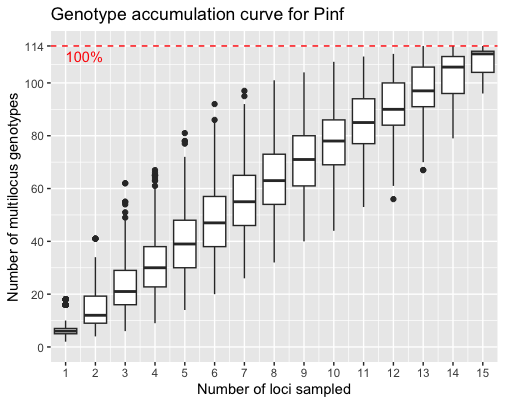


Fig. S1: Accumulation curve analysis for SSR markers to assess their suitability for analyzing the *Pst* population.
